# Clofibrate treatment in pigs: Effects on parameters critical with respect to peroxisome proliferator-induced hepatocarcinogenesis in rodents

**DOI:** 10.1186/1471-2210-7-6

**Published:** 2007-04-16

**Authors:** Sebastian Luci, Beatrice Giemsa, Gerd Hause, Holger Kluge, Klaus Eder

**Affiliations:** 1 Institut für Agrar- und Ernährungswissenschaften, Martin-Luther-Universität Halle-Wittenberg, Emil-Abderhalden-Strasse 26, D-06108 Halle (Saale), Germany; 2 Biozentrum, Martin-Luther-Universität Halle-Wittenberg, Weinbergweg 22, 06120 Halle (Saale), Germany

## Abstract

**Background:**

In rodents treatment with fibrates causes hepatocarcinogenesis, probably as a result of oxidative stress and an impaired balance between apoptosis and cell proliferation in the liver. There is some debate whether fibrates could also induce liver cancer in species not responsive to peroxisome proliferation. In this study the effect of clofibrate treatment on peroxisome proliferation, production of oxidative stress, gene expression of pro- and anti-apoptotic genes and proto-oncogenes was investigated in the liver of pigs, a non-proliferating species.

**Results:**

Pigs treated with clofibrate had heavier livers (+16%), higher peroxisome counts (+61%), higher mRNA concentration of acyl-CoA oxidase (+66%), a higher activity of catalase (+41%) but lower concentrations of hydrogen peroxide (-32%) in the liver than control pigs (P < 0.05); concentrations of lipid peroxidation products (thiobarbituric acid-reactive substances, conjugated dienes) and total and reduced glutathione in the liver did not differ between both groups. Clofibrate treated pigs also had higher hepatic mRNA concentrations of bax and the proto-oncogenes c-myc and c-jun and a lower mRNA concentration of bcl-X_L _than control pigs (P < 0.05).

**Conclusion:**

The data of this study show that clofibrate treatment induces moderate peroxisome proliferation but does not cause oxidative stress in the liver of pigs. Gene expression analysis indicates that clofibrate treatment did not inhibit but rather stimulated apoptosis in the liver of these animals. It is also shown that clofibrate increases the expression of the proto-oncogenes c-myc and c-jun in the liver, an event which could be critical with respect to carcinogenesis. As the extent of peroxisome proliferation by clofibrate was similar to that observed in humans, the pig can be regarded as a useful model for investigating the effects of peroxisome proliferators on liver function and hepatocarcinogenesis.

## Background

Peroxisome proliferators (PPs) comprise a diverse group of chemicals, including pharmaceuticals, industrial chemicals, endogenous fatty acids and eicosanoids. They bind to and activate the peroxisome proliferator-activated receptor (PPAR)-α, a transcription factor belonging to the nuclear hormone receptor superfamily [1]. Activation of PPARα causes an increase in the transcription of genes related to fatty acid transport across the cell membrane, intracellular lipid trafficking, mitochondrial and peroxisomal fatty acid uptake, and both mitochondrial and peroxisomal fatty acid β-oxidation [2]. Administration of PPs to rats and mice typically causes hepatic peroxisome proliferation, hypertrophy, hyperplasia, and hepatocarcinogenesis [3,4]. PPARα-induced hepatocarcinogenesis in rats and mice may be mainly due to an increased oxidative stress caused by peroxisome proliferation and an alteration of the balance between apoptosis and cell proliferation [5,6]. Treatment with PPs such as fibrates causes a 15 to 20-fold up-regulation of acyl-CoA oxidase (ACO) and other peroxisomal oxidases that lead to the production of hydrogen peroxide (H_2_O_2_) which under normal non-induced circumstances can be detoxified by catalase [7,8]. Catalase induction increases only approximately twofold in response to PPARα agonists in rodents, and the activity of glutathione peroxidase is often depressed following long term administration of PPs [9,10]. The capacity of the H_2_O_2_-degrading enzymes therefore may be insufficient to detoxify the large increase in H_2_O_2_. Increased cellular H_2_O_2 _could also react with metals and generate highly reactive hydroxyl radicals that could damage DNA, proteins or lipids [7]. Indeed, oxidatively damaged DNA and peroxide-modified lipids have been found in hepatocytes of rats treated with PPs [11-13]. Activation of PPARα also leads to increases in hepatocellular proliferation and inhibition of apoptosis, and when this occurs in DNA-damaged cells, it is thought to lead to proliferation of initiated cells progressing to liver tumour [14,15]. That PPARα is required to mediate hepatocarcinogenesis by PPs has been demonstrated in studies with PPARα-null mice that are refractory to this in response to long term administration of PPs [15,16].

It is well known that non-human primates and humans are only weakly responsive to peroxisome proliferation in comparison to rodents [17,18]. Nevertheless, there is considerable controversy as to whether the administration of drugs which are ligands for PPARα to humans causes liver cancer. This is significant because PPARα agonists such as fibrates have been in clinical use for the treatment of hyperlipidaemias for many years. The data regarding the ability of fibrates to cause peroxisome proliferation in humans are diverse. Examination of liver biopsy samples from patients receiving therapeutic doses of PPARα agonists showed a slight increase in peroxisome counts [19], while others showed no increase [20,21]. In non-human primates, administration of clofibrate induced a moderate, dose-dependent peroxisome proliferation [22-24]. It is therefore conceivable that cellular events induced by PPs related to hepatocarcinogenesis in rodents could occur also in non-proliferating species, albeit probably less pronounced than in rodents.

Pigs, like humans and non-human primates, are a non-proliferating species [25]. They may therefore be a valuable model for investigating the effects of PPs on peroxisome proliferation and related processes. To our knowledge, the effect of fibrates on parameters related to hepatocarcinogenesis has not yet been investigated in pigs. We therefore treated pigs with clofibrate and determined hepatic weight, number of peroxisomes and ACO expression to provide information about the potency of fibrates to induce peroxisome proliferation in pigs. We also considered the antioxidant status of the pigs (mRNA concentrations and activities of various antioxidant enzymes including catalase, generation of H_2_O_2 _in the liver, concentrations of lipid peroxidation products) to find out whether clofibrate causes oxidative stress in pig liver. In order to ascertain whether clofibrate treatment could affect the balance between cell proliferation and apoptosis we determined mRNA concentrations of pro- and anti-apoptotic genes, namely bax, bcl-X_L _and p53 tumour suppressor gene. It has been shown that the nuclear factor κB (NF-κB) pathway is important in the activation of genes that regulate cell proliferation and apoptosis in various cell types [26,27]. It was shown recently that NF-κB contributes to the proliferative and apoptotic changes that occur in liver in response to fibrates [28]. More recently, it has been demonstrated that the p50 subunit of the NF-KB family is necessary for the promotion of hepatocarcinogenesis by PPs [29]. To find out whether clofibrate treatment activated NF-KB in pig liver, we determined the mRNA concentration of tumor necrosis factor α (TNFα), a target gene of NF-KB which has also been identified as a suppressor of apoptosis and an inducer of DNA synthesis [30,31]. In rat liver and in mouse liver epithelial cells, treatment with the PPARα agonist WY-14,643 strongly up-regulated gene expression of various proto-oncogenes including c-fos, c-jun and c-myc [32-35]. In mouse liver cells these changes were followed by enhanced DNA synthesis, and it has been concluded that this could play an important role in tumour promotion by PPs [33]. Whether PPs stimulate expression of proto-oncogenes in pigs has not yet been investigated. We therefore also determined gene expression of c-myc, c-jun and c-fos in liver of pigs treated with clofibrate, which could be critical with respect to hepatocarcinogenesis.

## Results

Due to the controlled feeding system, diet intake during the whole experimental period was identical in both groups of pigs, being 696 ± 7 g/d in average of the whole period. Final body weights of the pigs on day 29 did not differ between the control group and the group treated with clofibrate (26.0 ± 1.5 kg for control pigs *vs*. 25.2 ± 1.2 kg for pigs treated with clofibrate, *n *= 9 in each group). Pigs treated with clofibrate had higher liver weights (+16% in absolute terms, +19% in relative terms, expressed per kg body weight), higher peroxisome counts in the liver (+61%) (P < 0.05, Table 1). Relative mRNA concentration of PPARα in liver did not differ between both groups of pigs (control: 1.00 ± 0.38; clofibrate: 0.92 ± 0.20, n = 9, means ± SD) but pigs treated with clofibrate had a higher relative mRNA concentration of ACO in the liver (+66%) than control pigs (P < 0.05, Table 1).

**Table 1:** Liver weights, number of peroxisomes and relative acyl-CoA oxidase mRNA concentration in the liver of pigs fed a control diet or a diet supplemented with 5 g clofibrate per kg for 28 days

Treatment	Control (*n *= 9)	Clofibrate (*n *= 9)
Liver weight (g)	673 ± 63	779 ± 63*
Liver weight (g/kg body weight)	25.9 ± 2.2	30.9 ± 2.6*
Number of peroxisomes (n/1,000 print)	366 ± 67	590 ± 116*
Acyl-CoA oxidase mRNA	1.00 ± 0.35	1.66 ± 0.41*

Results are means ± SD.

*Significantly different from control group (P < 0.05)

As reference values for the expression of enzyme activities, we determined concentrations of protein in liver homogenate and liver cytosol. Protein concentration in liver homogenate did not differ between both groups of pigs (control: 20.8 ± 5.9 mg/g liver; clofibrate: 21.1 ± 5.8 mg/g liver, n = 9, means ± SD); protein concentration in liver cytosol was higher in pigs treated with clofibrate than in control pigs (control: 27.2 ± 2.3 mg/g liver; clofibrate: 30.4 ± 3.0 mg/g liver, n = 9, means ± SD, P < 0.05). Pigs treated with clofibrate had higher mRNA concentrations and activities of superoxide dismutase (SOD) (+77% and +128%, respectively) and catalase (+72% and +41%, respectively) in the liver than control pigs (P < 0.05, Table 2). In contrast, mRNA concentration and activity of glutathione peroxidase (GSH-Px) in liver were reduced by 26% and 15%, respectively, in pigs treated with clofibrate compared to control pigs (P < 0.05, Table 2). mRNA concentration and activity of glutathione S-transferase (GST) in liver cytosol and concentrations of total and reduced glutathione in liver homogenate did not differ between both groups of pigs (Table 2). However, the concentration of α-tocopherol, both in absolute terms and in relative terms, expressed per mmol of triglycerides + total cholesterol, was lower in the liver of pigs treated with clofibrate than in the liver of control pigs (-40%, P < 0.05) (Table 2).

**Table 2:** Relative mRNA concentrations and activities of antioxidant enzymes and concentrations of some antioxidants in the liver of pigs fed a control diet or a diet supplemented with 5 g clofibrate per kg for 28 days

Treatment	Control (*n *= 9)	Clofibrate (*n *= 9)
Catalase		
mRNA concentration	1.00 ± 0.49	1.72 ± 0.58*
Activity (U/mg protein)	0.75 ± 0.14	1.06 ± 0.16*
Glutathione peroxidase		
mRNA concentration	1.00 ± 0.17	0.74 ± 0.18*
Activity (U/mg protein)	4.7 ± 0.8	4.0 ± 0.4*
Glutathione S-transferase		
mRNA concentration	1.00 ± 0.41	0.90 ± 0.32
Activity (U/mg protein)	0.76 ± 0.26	0.76 ± 0.18
Superoxide dismutase		
mRNA concentration	1.00 ± 0.42	1.77 ± 0.40*
Activity (U/mg protein)	43 ± 8	97 ± 13*
Glutathione, total (nmol/mg protein)	2.13 ± 0.51	1.97 ± 0.90
Glutathione, reduced (nmol/mg protein)	1.70 ± 0.50	1.67 ± 1.02
α-tocopherol		
(nmol/g)	14.5 ± 2.5	8.7 ± 2.9*
[nmol/(mmol triglycerides + cholesterol)]	2.32 ± 0.40	1.36 ± 0.45*

Results are means ± SD.

*Significantly different from control group (P < 0.05)

^#^Relative to control (= 1.00)

The concentration of H_2_O_2 _in the liver was about 32% lower in pigs treated with clofibrate than in control pigs (P < 0.05, Table 3). Concentrations of lipid peroxidation products, thiobarbituric acid-reactive substances (TBARS) and conjugated dienes did not differ between both groups of pigs, both in absolute terms and in relative terms, expressed per mmol of triglycerides + total cholesterol (Table 3).

**Table 3:** Concentration of hydrogen peroxide and lipid peroxidation products in the liver of pigs fed a control diet or a diet supplemented with 5 g clofibrate per kg for 28 days

Treatment	Control (*n *= 9)	Clofibrate (*n *= 9)
Hydrogen peroxide (fluorescence/g liver)	29,437 ± 8,361	20,078 ± 7,225*
Thiobarbituric acid-reactive substances		
(nmol/g liver)	7.2 ± 1.6	7.7 ± 2.7
[nmol/(mmol triglycerides + cholesterol)]	1.15 ± 0.26	1.20 ± 0.42
Conjugated dienes (nmol/g)		
(nmol/g liver)	16 ± 2	16 ± 2
[nmol/(mmol triglycerides + cholesterol)]	2.56 ± 0.32	2.49 ± 0.31

Results are means ± SD.

*Significantly different from control group (P < 0.05)

**Figure 1:**
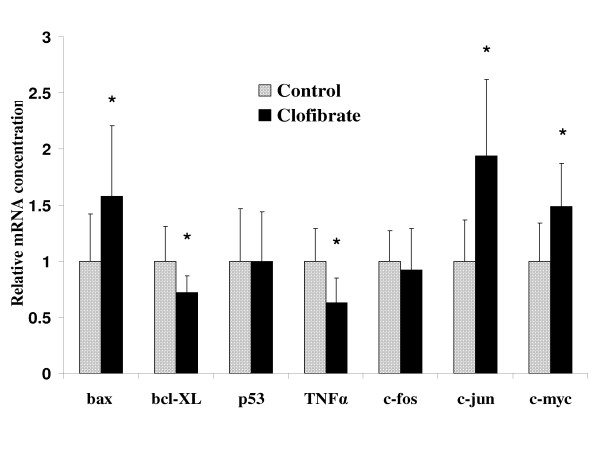
Relative mRNA concentrations of pro- and anti-apoptotic genes (bax, bcl-X_L_, p53, TNFα) and proto-oncogenes (c-myc, c-jun, c-fos) in livers of pigs fed a control diet or a diet supplemented with 5 g clofibrate per kg for 28 days. All mRNA concentrations were determined by real-time quantitative PCR and normalized to GAPDH. Data are reported as means ± SD with nine animals per group. Data are expressed relative to mRNA concentrations of control pigs (control = 1). * Significantly different to control group (P < 0.05).

Hepatic mRNA concentrations of p53 and c-fos did not differ between pigs treated with clofibrate and control pigs whereas mRNA concentrations of bax, c-jun and c-myc were higher in pigs treated with clofibrate than in control pigs (P < 0.05, Fig. 1). Hepatic mRNA concentrations of bcl-X_L _and TNFα were lower in pigs treated with clofibrate than in control pigs (P < 0.05, Fig. 1).

## Discussion

To our knowledge, this is the first study to investigate the effect of clofibrate treatment on peroxisome proliferation and parameters that may be related to hepatocarcinogenesis in pigs, a non-proliferating species. As in many other studies dealing with the effects of clofibrate on metabolism in experimental animals, we added clofibrate to the diet. The concentration of clofibrate in the diet of 5 g per kg diet was adopted from other studies with pigs [25,36,37]. The resulting daily dose of 220 mg per kg body weight was relatively high compared with doses used in humans for treatment of hyperlipidaemia, which are usually in the range between 25 and 30 mg per kg body weight.

Analysis of liver weights and number of peroxisomes showed that treatment with clofibrate caused moderate peroxisome proliferation in pigs. The increase in the number of peroxisomes (+62%) observed in pigs treated with clofibrate is of a similar order of magnitude as the 50% increase in liver peroxisome counts observed in humans treated with clofibrate [19]. The extent of the change in peroxisome counts in pigs is modest when compared with that reported for rodents. In rodents, administration of a dose of 200 mg clofibrate per kg body weight, comparable with that used in this study in pigs, resulted in a three- to five-fold increase in peroxisome counts compared with controls [38]. The current study also shows that gene expression of ACO is moderately increased by clofibrate in pigs, which is in close accord with the moderate effect of clofibrate on the peroxisome count. The present study confirms other studies [25,36,37] which have also shown that clofibrate causes only a relatively weak up-regulation of PPARα target genes in pig liver. The moderate effect of clofibrate on ACO in pigs is in strong contrast to rodents where treatment with PPs causes a 10 to 20-fold increase in ACO expression [35,39]. Up-regulation of ACO in the liver is critical because it leads to increased production of H_2_O_2 _which causes oxidative stress within the cell. In contrast to observations in rodents, pigs treated with clofibrate in this study had not a higher but a lower concentration of H_2_O_2 _in the liver. We assume that this is due to up-regulation of catalase, the key enzyme involved in H_2_O_2 _detoxification. In our study both ACO and catalase were up-regulated by clofibrate to a similar extent. Catalase has a high H_2_O_2_-detoxifying activity and is the rate-limiting enzyme for inhibiting H_2_O_2 _leakage from peroxisomes [40]. The finding that the concentration of H_2_O_2 _was reduced even though the activity of GSH-Px, another H_2_O_2_-detoxifying enzyme localised in cytosol was reduced as well, suggests that the up-regulation of catalase was sufficient to eliminate all of the H_2_O_2 _produced in peroxisomes. The finding of a reduced activity of GSH-Px in liver of pigs treated with clofibrate agrees with findings in rodents treated with PPs which also showed a lower activity of that enzyme in the liver [11,41].

Unsaturated fatty acids are susceptible to reactive oxygen species and undergo oxidation. The determination of lipid peroxides such as TBARS or conjugated dienes is therefore a sensible method to detect oxidative stress. Indeed, in hepatocytes of rats treated with PPs, concentrations of lipid peroxidation products were increased due to oxidative stress induced by peroxisome proliferation [11,42]. The fact that concentrations of TBARS and conjugated dienes were not increased in the liver of pigs treated with clofibrate indicates that the moderate peroxisome proliferation was not accompanied by oxidative stress. This indication is supported by the observation that the concentration of reduced glutathione in the liver was also not altered in pigs treated with clofibrate when compared with control pigs. Glutathione plays a pivotal role in protecting cells against the noxious effects of oxidant agents, and oxidative stress leads to enhanced oxidation of glutathione, which in turn causes a lower concentration of reduced glutathione and a lower ratio of reduced to oxidized glutathione [43]. Likewise, activity of GST, an enzyme belonging to the phase II enzymes whose reactions include the addition of glutathione to electrophilic molecules as well as the detoxification of organic hydroperoxides, was not changed in pigs treated with clofibrate. This is in contrast to rodents treated with fibrates or other PPs that have a strongly reduced activity of GST in the liver [44,45]. As it has been suggested that the reduced GST activity in rodents treated with PPs is the consequence of oxidative stress due to peroxisome proliferation, the finding of an unchanged activity of that enzyme is another indication that oxidative stress did not occur in pigs treated with clofibrate. This indication agrees with a recent study which investigated the effect of high doses of fenofibrate and ciprofibrate in cynomolgus monkeys [24]. In that study, clofibrate treatment induced moderate hepatic peroxisome proliferation, similar to that observed in the pigs of the present study, but there was also minimal indication of oxidative stress. It is therefore concluded that even high doses of fibrates cause little oxidative stress in the liver of non-proliferating species.

In the current study, pigs treated with clofibrate had increased hepatic mRNA concentration and activity of SOD, an enzyme that converts superoxide anions into H_2_O_2 _and therefore contributes to increased H_2_O_2 _production. The finding that H_2_O_2 _concentration in the liver was reduced in spite of the increased activity of SOD is another indication that pigs treated with fibrate had a high hepatic H_2_O_2_-detoxifying capacity. Results from other studies dealing with the effects of PPs on hepatic SOD are contradictory. In some studies, treatment of rodents with PPs lowered hepatic activity of SOD [41,46]; in others treatment with PPs increased the activity of hepatic SOD [47,48]. Moreover, it has been shown that different PPs can have different effects on hepatic activity of SOD, and that there are also differences between mice and hamsters in the effect of PPs on SOD activity [48]. The reason for these contradictory results is unclear and should be investigated further. As SOD is an important constituent of the hepatic antioxidant system, an increased activity of that enzyme observed in pigs treated with clofibrate could contribute to a high antioxidant capacity in the liver of these pigs.

The finding that pigs treated with clofibrate had a reduced concentration of α-tocopherol in the liver agrees with those of several other studies in which rodents were treated with PPs [11,48,49]. It has been shown that the tocopherol-lowering effect of PPs is stronger in rats than in hamsters, suggesting that there is a correlation with the degree of peroxisome proliferation [48]. It has been suggested that the reduction of hepatic tocopherol concentration is not primarily due to oxidative stress produced by PPs but rather to their hypolipidaemic effect [48]. Tocopherols are transported by lipoproteins within the body and as hypolipidaemic drugs reduce the number of lipoprotein particles in blood, they could also impair vitamin E transport in the body, i.e. transport of vitamin E from the intestine to the liver by chylomicrons. The fact that α-tocopherol concentration was reduced in the liver of pigs treated with clofibrate although there were no signs of oxidative stress confirms the suggestion that PPs do reduce hepatic vitamin E concentrations independent of oxidative stress.

It has been shown that peroxisome proliferation leads to hepatocellular proliferation and to inhibition of apoptosis, and these processes may contribute to hepatomegaly and to hepatocarcinogenesis observed in rodents treated with PPs [14,15]. The present study shows that clofibrate treatment of pigs increases gene expression of the proto-oncogenes c-jun and c-myc, which are required for entry into the S phase of the cell cycle. These findings are in agreement with recent studies in rodent livers and liver cells in which treatment with WY-14,643 strongly up-regulated gene expression of proto-oncogenes [32-34]. Up-regulation of proto-oncogenes in mouse liver cells was followed by enhanced progression, and it has been suggested that this could play a role in tumour promotion of PPs [32]. Although up-regulation of proto-oncogenes was smaller in pigs treated with fibrates than in rodents [32-34], increased levels of c-jun and c-myc could have enhanced cell proliferation, which might explain the increased liver mass in pigs treated with clofibrate. Up-regulation of proto-oncogenes could be a critical event with respect to tumorigenesis. On the other hand, c-myc can collaborate with other proteins to induce apoptosis and sensitize cells to a variety of apoptotic triggers [50,51]. In order to find out whether clofibrate treatment in pigs could have altered apoptosis, we determined mRNA concentrations of bax, bcl-X_L _and p53 in the liver. Genes of the bcl-2 family have anti-apoptotic effects, which is antagonized by bax. The bcl-2/bax ratio is a key factor for determining apoptosis. When bcl-2 is expressed excessively, bcl-2-bax heterogenous dimer predominates, thus inhibiting apoptosis [52]. When bax is expressed excessively, bax-bax homogenous dimer or monomer predominates, thus promoting apoptosis. p53 is a tumour suppressor which exerts control over cell cycling by controlling the progression through the G1 phase [53]. Genotoxic stress or DNA damage leads to nuclear accumulation of p53, which in turn activates the transcription of several genes involved in DNA repair or apoptosis, including bcl-2 [54]. We did not directly determine apoptosis in the liver of piglets but the finding that expression of bax was up-regulated in pigs treated with clofibrate while expression of bcl-X_L _was reduced, together with the observation of unchanged p53 expression suggests that apoptosis was not inhibited but could instead have been increased in these pigs. This suggestion agrees with recent studies which have shown that clofibrate induces apoptosis in human and rat hepatoma cells [55-59]. In agreement with our study, a recent study showed that treatment with WY 14,643 up-regulates pro-apoptotic genes and down regulates anti-apoptotic genes in the liver of mice, an effect which did not occur in PPARα-null mice [60]. In that study, it was also demonstrated that PPARα activation increases the sensitivity of liver towards apoptosis by Jo-2, an inducer of hepatic apoptosis. Therefore, it has been suggested that PPARα could serve as a pharmacological target in diseases where apoptosis is a contributing feature [60,61].

In a recent study in rats it was found that NF-KB is activated by WY-14,643, probably due to oxidative stress caused by peroxisome proliferation [62]. It has been demonstrated that NF-KB is essential for inducing cell proliferation and hepatocarcinogenesis in rodents treated with fibrates [28,29]. The finding that mRNA concentration of TNFα, a target gene of NF-κB, was reduced in liver of pigs treated with clofibrate compared to control pigs indicates that clofibrate did not activate the NF-KB pathway in these animals. This finding may be related to the observation that clofibrate treatment did not cause oxidative stress in pigs, in contrast to rats. The finding that WY-14,643 did not activate the NF-KB pathway in hamsters either [62] suggests that PPs activate NF-KB only in species that are responsive to PP induced hepatocarcinogenesis.

## Conclusion

Treatment with clofibrate at doses higher than those used for hypolipidaemic treatment in humans causes moderate peroxisome proliferation in the liver of pigs. Determination of the concentration of H_2_O_2 _and lipid peroxidation products indicates that this did not produce oxidative stress. Determination of mRNA concentrations of pro- and anti-apoptotic genes in the liver indicates that clofibrate treatment did also not inhibit but rather stimulated apoptosis in these animals. Up-regulation of the proto-oncogenes c-myc and c-jun in the liver, however, could be a critical event with respect to carcinogenesis, which deserves further investigation in future studies. As the extent of peroxisome proliferation by clofibrate was similar to that observed in humans, the pig can be regarded as a useful model for the investigating the effects of PPs on liver function and hepatocarcinogenesis.

## Methods

### Animals and treatments

Eighteen male 8-week-old crossbred pigs [(German Landrace × Large White) × Pietrain] were kept in a room under controlled conditions at 23 ± 2°C and 55 ± 5% relative humidity with light from 0600 to 1800 h. One day before the start of the experimental feeding period the pigs were weighed and randomly assigned to two groups with body weights of 12.0 ± 1.1 kg in the control group and 11.9 ± 0.6 kg in the treatment group. Both groups of pigs received a nutritionally adequate dry diet for growing pigs (according to [63]) containing (in g/kg) wheat (400), soybean meal (230), wheat bran (150), barley (100), sunflower oil (90) and mineral premix including L-lysine, DL-methionine and L-threonine (30). This diet contained 14.4 MJ metabolizable energy and 185 g crude protein per kg. The whole daily amount of diet was administered once at 8.00 h. Diet intake was controlled, and each animal in the experiment was offered an identical amount of diet per day. The amount of diet administered was about 15% below that consumed ad-libitum by pigs of a similar weight (as assessed in a previous study). Therefore, the diet offered was completely taken up by all pigs in the experiment. During feeding period, the amount of diet offered each day was increased continuously from 400 to 1,200 g. Pigs of both groups received the same diet. However, pigs of the treatment group were given additionally 5 g clofibrate per kg diet which was freshly given onto the diet on each day. The pigs had free access to water via nipple drinking systems. The experimental diets were administered for 28 d. All experimental procedures described followed established guidelines for the care and use of laboratory animals and were approved by the local veterinary office.

### Sample collection

After completion of the feeding period the piglets were captive-bolt stunned and exsanguinated. Four hours before euthanasia each pig was fed its respective diet. After killing, blood was collected into heparinized polyethylene tubes. Plasma was obtained by centrifugation of the blood (1,100 × g; 10 min; 4°C). The liver was dissected and weighed and samples were stored at -80°C until analysis. For preparation of liver homogenate, one g of liver was homogenized in 10 mL of 0.1 M phosphate buffer, pH 7.4, containing 0.25 M sucrose using a Potter-Elvehjem homogenizer. Homogenates were centrifuged at 1,000 × g for 10 min at 4°C and the supernatant was centrifuged at 15,000 × g for 15 min again. The supernatant of that centrifugation was collected and centrifuged at 105,000 × g for 60 min to yield the cytosolic fraction. Liver homogenates and cytosolic fraction were stored at -20°C for further analysis. Protein concentrations of liver homogenates and cytosol were determined with the bicinchoninic acid reagent according to the supplier's protocol (Interchim, Montelucon, France) using bovine serum albumin as the standard.

### RT-PCR analysis

Total RNA from liver tissue was isolated by TRIzol reagent (Invitrogen, Karlsruhe, Germany) following the manufacturer's protocol, resuspended in diethyl pyrocarbonate-treated water and stored at -80°C until use. The concentration and purity of total RNA was determined by ultraviolet absorbance at 260 and 280 nm (SpectraFluor Plus; Tecan, Crailsheim, Germany). The quality of all RNA samples was assessed by agarose gel electrophoresis. cDNA was prepared from total RNA (1.2 μg) by reverse transcription using M-MuLV Reverse Transcriptase (MBI Fermentas, St. Leon-Rot, Germany) and oligo(dT)_18 _primers (Operon Biotechnologies, Cologne, Germany). The mRNA concentration of genes was measured by realtime detection PCR using SYBR^® ^Green I and a MJ Research Opticon system (Biozym Diagnostik GmbH, Oldendorf, Germany). Realtime detection PCR was performed with 1.25 U Taq DNA polymerase (Promega, Mannheim, Germany), 500 μM dNTPs and 26.7 pmol of the specific primers (Operon Biotechnologies, Cologne, Germany; Table 4). Annealing temperature for all primers was 60°C. Amplification efficiencies for all primer pairs were determined by template dilution series. Calculation of the relative mRNA concentration was made using the amplification efficiencies and the C_t _values [64]. Glyceraldehyde 3-phosphate dehydrogenase (GAPDH) was used as housekeeping gene for normalization.

**Table 4:** Sequences of specific primers used for RT-PCR

Gene (NCBI Genbank)	Forward Primer	Reverse Primer	Size, bp
ACO (AF185048)	CTCGCAGACCCAGATGAAAT	TCCAAGCCTCGAAGATGAGT	218
bax (AJ606301)	CGAACTGATCAGGACCATCA	ACAGCCCATCTTCTTCCAGA	190
bcl-X_L _(NM_214285)	GAAACCCCTAGTGCCATCAA	GGGACGTCAGGTCACTGAAT	196
Catalase (NM_214301)	CAGCTTTAGTGCTCCCGAAC	AGATGACCCGCAATGTTCTC	180
c-fos (Y14808)	CTGACACACTCCAAGCGGTA	CTTCTCCTTCAGGTTGG	209
c-jun (NM_213880)	CAGAGCATGACCCTGAACCT	TTCTTGGGGCATAGGAACTG	200
c-myc (NM_001005154)	AATGTCTTGGAACGCCAGAG	CAACTGTTCTCGCCTCTTCC	204
GAPDH (AF017079)	AGGGGCTCTCCAGAACATCATCC	TCGCGTGCTCTTGCTGGGGTTGG	446
GSH-Px (NM_214201)	CAAGAATGGGGAGATCCTGA	GATAAACTTGGGGTCGGTCA	190
GST (NM_214300)	TTTTTGCCAACCCAGAAGAC	GGGGTGTCAAATACGCAATC	246
p53 (NM_214145)	GCGAGTATTTCACCCTCCAG	TCAGGCCCTTCTCTCTTGAA	199
PPARα (DQ437887)	CAGCCTCCAGCCCCTCGTC	GCGGTCTCGGCATCTTCTAGG	381
SOD (AF396674)	TCCATGTCCATCAGTTTGGA	CTGCCCAAGTCATCTGGTTT	250
TNFα (X57321)	CCCCTGTCCATCCCTTTATT	AAGCCCCAGTTCCAATTCTT	200

Abbreviations: ACO, acyl-CoA oxidase; GAPDH, glyceraldehyde-3-phosphate dehydrogenase; GSH-Px, glutathione peroxidase; GST, glutathione S-transferase; SOD, superoxide dismutase; TNFα, tumor necrosis factor α.

### Enzyme assays

SOD in liver cytosol was determined with pyrogallol as the substrate [65]. One unit of SOD activity is defined as the amount of enzyme required to inhibit the autoxidation of pyrogallol by 50%. The activity of GSH-Px in liver cytosol was determined with t-butyl hydroperoxide as substrate [66]. One unit of GSH-Px activity is defined as one μmol reduced β-nicotinamide adenine dinucleotide phosphate oxidized per min. The activity of GST was determined using 1-chloro-2,4-dinitrobenzene as substrate [67]. One unit of GST is defined as one nmol substrate consumed per min. Catalase activity in liver homogenate was determined using hydrogen peroxide as substrate [68]. One unit of catalase activity is defined as the amount consuming 1 mmol hydrogen peroxide per min. GSH concentration in liver homogenates was determined according to Griffith [69].

### Determination of conjugated dienes, TBARS, α-tocopherol, cholesterol and triglycerides in the liver

Lipids from liver were extracted using a mixture of n-hexane and isopropanol (3:2, v/v). After drying the lipid extracts, 1 mg of extract was dissolved in 1 ml n-hexane. The concentrations of conjugated dienes were calculated by using the molar extinction coefficient for conjugated dienes at 234 nm (ε = 29,500 mol × cm^-1^). The concentrations of TBARS were measured with thiobarbituric acid as reagent in a fluorimetric assay [70]. Concentration of α-tocopherol in liver tissue was determined by HPLC with fluorescence detection [70]. For determination of triglycerides and total cholesterol, an aliquot of the lipid extract was dried, and the dried lipids were dissolved with Triton X-100 [71]. The concentrations of cholesterol and triglycerides were determined using enzymatic kits (Cat.-No. 113009990314 for cholesterol and Cat.-No. 157609990314 for triglycerides, Ecoline S^+^, DiaSys, Holzheim, Germany).

### Determination of H_2_O_2_

To determine the H_2_O_2 _content in liver homogenates, a method [72] described for cell culture systems was modified, using dihydrorhodamine 123 (DHR) as substrate. Homogenates were incubated with 27.5 μM DHR for 1 h at 37°C in a final volume of 400 μl. After incubation, the fluorescence of rhodamine 123, the oxidation product of DHR, was measured (excitation wavelength: 485 nm, emission wavelength: 538 nm). As previously shown [73], this test is specific for H_2_O_2 _as DHR is specifially oxidized by H_2_O_2_.

### Transmission electron microscopy

Small liver segments of three animals per group were fixed immediately after dissection of the liver with 3% glutaraldehyde in 0.1 M sodium cacodylate buffer (SCB, pH 7.2) for 3 hours at room temperature, washed with SCB, postfixed with 1% osmiumtetroxide in SCB, dehydrated in a graded ethanol series, and embedded in epoxy resin [74]. The material was sectioned with an ultramicrotome S (Leica, Bensheim, Germany). Ultrathin sections (80 nm) were transferred to coated copper grids and poststained with uranyl acetate and lead citrate. The sections were observed with an EM 900 transmission electron microscope (Zeiss SMT, Oberkochen, Germany) at an acceleration voltage of 80 kV. Electron micrographs were taken with a slow scan camera (TRS, Dünzelbach, Germany). Peroxisomes were counted in 1,000 different regions per liver sample for each animal at a screen (12,000-fold magnification).

### Statistics

The results were analyzed using Minitab (State College, Pa, USA) statistical software (release 13). Statistical significance of differences between control group and treatment group was evaluated using Student's t-test. Mean values were considered significantly different for P < 0.05.

## List of abbreviations

ACO, acyl-CoA oxidase; DHR, dihydrorhodamine; GAPDH, glyceraldehyde 3-phosphate dehydrogenase; GSH-Px, glutathione peroxidase; GST, glutathione S-transferase; NF-KB, nuclear factor KB; PPARα, peroxisome proliferator-activated receptor α; PPs, peroxisome proliferators; SCB, sodium cacodylate buffer; SOD, superoxide dismutase; TBARS, thiobarbituric acid-reactive substances; TNFα, tumor necrosis factor α.

## Authors' contributions

SL and BG carried out the feeding experiments, performed the analyses and helped to draft the manuscript.

GH determined peroxisome count in the liver by transmission electrone microscopy.

HK participated in the design of the study and in the interpretation of the results and supervised the animal experiment.

KE conceived the study and its design, coordinated work, participated in the interpretation of the results, and prepared the manuscript.

All authors read and approved the final manuscript.
